# Supplementary material for: Quantitative Risk Assessment of Susceptible and Ciprofloxacin-Resistant Salmonella from Retail Pork in Chiang Mai Province in Northern Thailand
Source: Foods. 2022 Sep 20;11(19):2942. doi: 10.3390/foods11192942 (PMC9562186; doi:10.3390/foods11192942)
Supplement: Supplementary file 1 [file foods-11-02942-s001.zip › foods-1841355-supplementary.pdf]

1. PCR detection of carbapenemase genes

The primers used in the PCR are listed in Table S1 [1, 2]. The reaction mixture (25 µl) contained 1X JumpStart™ REDTaq® ReadyMix™ Reaction Mix (Sigma-Aldrich), 0.4 µM of each primer, sterile deionized (DI) water, and bacterial DNA template. The amplification conditions were as follows: initial denaturation at 95 °C for 3 min, followed by 30 cycles of denaturation at 95 °C for 30 s, annealing at 58 °C for 45 s, and extension at 72 °C for 1 min, and a final extension at 72 °C for 7 min. PCR products were evaluated on a 2% agarose gel at 100 V for 30 min. The gel was stained with ethidium bromide, and the DNA bands were visualized and photographed under ultraviolet light using gel documentation system (Syngene, UK).

Table S1. Primers for carbapenemase genes

Primer	Sequence (5′-3′)	Gene	Product size (bp)
IMP-F	GGAATAGAGTGGCTTAAYTCTC	bla_{IMP}	232
IMP-R	GGTTTAAYAAAACAACCACC		
SPM-F	AAAATCTGGGTACGCAAACG	bla_{SPM}	271
SPM-R	ACATTATCCGCTGGAACAGG		
AIM-F	CTGAAGGTGTACGGAAACAC	bla_{AIM}	322
AIM-R	GTTCGGCCACCTCGAATTG		
VIM-F	GATGGTGTTTGGTCGCATA	bla_{VIM}	390
VIM-R	CGAATGCGCAGCACCAG		
OXA-F	GCGTGGTTAAGGATGAACAC	$bla_{OXA\text{-}48}$	438
OXA-R	CATCAAGTTCAACCCAACCG		
GIM-F	TCGACACACCTTGGTCTGAA	bla_{GIM}	477
GIM-R	AACTTCCAACTTTGCCATGC		
BIC-F	TATGCAGCTCCTTTAAGGGC	bla_{BIC}	537
BIC-R	TCATTGGCGGTGCCGTACAC		
SIM-F	TACAAGGGATTCGGCATCG	bla_{SIM}	570
SIM-R	TAATGGCCTGTTCCCATGTG		
NDM-F	GGTTTGGCGATCTGGTTTTC	bla_{NDM}	621
NDM-R	CGGAATGGCTCATCACGATC		
DIM-F	GCTTGTCTTCGCTTGCTAACG	bla_{DIM}	699
DIM-R	CGTTCGGCTGGATTGATTTG		
KPC-F	CGTCTAGTTCTGCTGTCTTG	bla_{KPC}	789
KPC-R	CTTGTCATCCTTGTTAGGCG		

2. PCR detection of plasmid-mediated colistin resistance genes (*mcr-1-mcr-9*)

Primers for mobilized colistin resistance genes (*mcr-1–mcr-9*) are shown in Table S3 [4]. The reaction mixture (25 µl) contained 1X JumpStart™ REDTaq® ReadyMix™ Reaction Mix (Sigma-Aldrich), 0.4 µM of each primer, sterile DI water, and bacterial DNA template. The amplification conditions were as follows: initial denaturation at 95 °C for 3 min, followed by 30 cycles of denaturation at 95 °C for 30 s, annealing at 62 °C for 75 s, and extension at 72 °C for 5 min. PCR products were separated on a 2% agarose gel at 100 V for 30 min. The gel was stained with ethidium bromide, and the DNA bands were visualized and photographed under ultraviolet light using gel documentation system (Syngene,UK).

Table S2. Primers for plasmid-mediated colistin resistance genes (*mcr-1-mcr-9*)

Primer	Sequence (5′-3′)	Gene	Product size (bp)
mcr-1F	TTGCCAAATTCACGCCAGTG	*mcr-1*	320
mcr-1R	CTTTGACTTTGTCCGCGGTG		
mcr-2F	CAAGTGTGTTGGTCGCAGTT	*mcr-2*	700
mcr-2R	TCTAGCCCGACAAGCATACC		
mcr-3F	CTGAACTGGCGTGGACGTTCT	*mcr-3*	1,200
mcr-3R	ATCATCCGGTGCAAACTGGT		
mcr-4F	TCACTTTCATCACTGCGTTG	*mcr-4*	1,150
mcr-4R	TTGGTCCATGACTACCAATG		
mcr-5F	ATGCGGTTGTCTTGCATTTATC	*mcr-5*	1,300
mcr-5R	TCATTGTGGTTGTCCTTTTCTG		
mcr-6F	GTCCGGCTCAATCCCTATCTGT	*mcr-6*	500
mcr-6R	ATCACGGGATTGACATAGCTAC		
mcr-7F	TGCTCAAGCCCTTCTTTTCGT	*mcr-7*	900
mcr-7R	TTCATCTGCGCCACCTCGT		
mcr-8F	GGGGGTAACCAACCCCTATC	*mcr-8*	250
mcr-8R	TGCCGGCATATCACTCGTGG		
mcr-9F	AGAACATGCACGGAACGGAT	*mcr-9*	190
mcr-9R	CTCACGAAAAACCCACCTG		

3. PCR detection of plasmid-mediated quinolone resistance (PMQR) genes

The PMQR primers are shown in Table S4 [5]. The reaction mixture (25 µl) contained 1X JumpStart™ REDTaq® ReadyMix™ Reaction Mix (Sigma-Aldrich), 0.25 µM of each primer, sterile DI water, and bacterial DNA template. The amplification conditions were as follows: initial denaturation at 95 °C for 15 min, followed by 30 cycles of denaturation at 95 °C for 30 s, annealing at 63 °C for 90 s, and extension at 72 °C for 90 s, and a final amplification at 72 °C for 10 min. PCR products were evaluated on 2% agarose gels at 100 V for 30 min. The gel was stained with ethidium bromide, and the DNA bands were visualized and photographed under ultraviolet light using gel documentation system (Syngene,UK).

Table S3. Primers for plasmid-mediated quinolone resistance (PMQR) genes

Primer	Sequence (5′-3′)	Gene	Product size (bp)
qnrA-F	CAGCAAGAGGATTTCTCACG	*qnrA*	630
qnrA-R	AATCCGGCAGCACTATTACTC		
qnrD -F	CGAGATCAATTTACGGGGAATA	*qnrD*	581
qnrD –R	AACAAGCTGAAGCGCCTG		
qnrB-F	GGCTGTCAGTTCTATGATCG	*qnrB*	488
qnrB-R	GAGCAACGATGCCTGGTAG		
qnrS-F	GCAAGTTCATTGAACAGGGT	*qnrS*	428
qnrS-R	TCTAAACCGTCGAGTTCGGCG		
oqxAB-F	CCGCACCGATAAATTAGTCC	*oqxAB*	313
oqxAB-R	GGCGAGGTTTTGATAGTGGA		
aac(6')-Ib-cr-F	TTGGAAGCGGGGACGGAM	*aac(6')-Ib-cr*	260
aac(6')-Ib-cr-R	ACACGGCTGGACCATA		
qepA-F	GCAGGTCCAGCAGCGGGTAG	*qepA*	218
qepA-R	CTTCCTGCCCGAGTATCGTG		
qnrC-F	GCAGAATTCAGGGGTGTGAT	*qnrC*	118
qnrC-R	AACTGCTCCAAAAGCTGCTC		

4. PCR detection of quinolone resistance-determining region (QRDR) genes

The QRDR primers used in PCR are shown in Table S5 [6]. The reaction was carried out in a final volume of 50 µl containing 1X JumpStart™ REDTaq® ReadyMix™ Reaction Mix (Sigma-Aldrich), 0.6 µM of each primer, sterile DI water, and bacterial DNA template. The amplification conditions were as follows: initial denaturation at 95 ºC for 5 min, followed by 30 cycles of denaturation at 95 ºC for 30 s, annealing at 53.4ºC for *gyrA, parC, marA* and 60 ºC for *gyrB*, for 90 s and extension at 72 ºC for 90 s, and a final amplification at 72 ºC for 10 min. PCR products were evaluated on a 2% agarose gel at 100 V for 30 min. The gel was stained with ethidium bromide, and the DNA bands were visualized and photographed under ultraviolet light by gel documentation system (Syngene,UK).

The PCR products of the above genes were purified using an E-Z 96 Cycle Pure Kit (Omega, Norcross, GA, USA) following the manufacturer's instructions. Sanger DNA sequencing of the purified PCR products was performed by Apical Scientific Sdn Bhd, Selangor, Malaysia.

Table S4. Primers for quinolone resistance-determining region (QRDR) genes

Primer	Sequence (5′-3′)	Gene	Product size (bp)
gyrA-F	TATGCGATGTCGGTCATTGT	*gyrA*	369
gyrA-R	CACGAAATCCACCGTCTCTT		
gyrB-F	GCGCTGTCCGAACTGTACCT	*gyrB*	172
gyrB-R	CGGTGATCAGCGTCGCCACTTCC		
parC-F	TGCGTTGCCGTTTATTGG	*parC*	303
parC-R	TCGGCGTATTTGGACAGG		
marA-F	GCAACGCTTGAGTATTTGCT	*marA*	479
marA-R	CATTTTCATGGTGCTCTTCG		

References

1. Poirel L, Walsh TR, Cuvillier V, Nordmann P. Multiplex PCR for detection of acquired carbapenemase genes. *Diagn Microbiol Infect Dis* 2011;70:119-123. doi: 10.1016/j.diagmicrobio.2010.12.002.

2. Hatrongjit R, Kerdsin A, Akeda Y, Hamada S. Detection of plasmid-mediated colistin-resistant and carbapenem-resistant genes by multiplex PCR. *MethodsX* 2018;5:532-536. doi.org/10.1016/j.mex.2018.05.016

3. Khanawapee A, Kerdsin A, Chopjitt P, Boueroy P, Hatrongjit R *et al.* Distribution and Molecular Characterization of *Escherichia coli* Harboring *mcr* Genes Isolated from Slaughtered Pigs in Thailand. *Microb Drug Resist* 2020 Dec 15. doi: 10.1089/mdr.2020.0242. In Press.

4. Ciesielczuk H, Hornsey M, Choi V, Woodford N, Wareham DW. Development and evaluation of a multiplex PCR for eight plasmid-mediated quinolone-resistance determinants. *J Med Microbiol* 2013;62:1823-1827. doi: 10.1099/jmm.0.064428-0.

5. Lu Y, Zhao H, Liu Y, Zhou X, Wang J *et al.* Characterization of quinolone resistance in *Salmonella enterica* serovar Indiana from chickens in China. *Poult Sci* 2015;94:454-460. doi.org/10.3382/ps/peu133
